# Coat Color Roan Shows Association with *KIT* Variants and No Evidence of Lethality in Icelandic Horses

**DOI:** 10.3390/genes11060680

**Published:** 2020-06-22

**Authors:** Katharina Voß, Julia Tetens, Georg Thaller, Doreen Becker

**Affiliations:** 1 Institute of Animal Breeding and Husbandry, University of Kiel, Olshausenstraße 40, 24098 Kiel, Germany; kvoss@tierzucht.uni-kiel.de (K.V.); juliatetens@tierzucht.uni-kiel.de (J.T.); gthaller@tierzucht.uni-kiel.de (G.T.); 2 Institute of Genome Biology, Leibniz Institute for Farm Animal Biology, Wilhelm-Stahl-Allee 2, 18196 Dummerstorf, Germany

**Keywords:** coat color, roan, Icelandic horse

## Abstract

Roan (Rn) horses show a typical seasonal change of color. Their body is covered with colored and white hair. We performed a descriptive statistical analysis of breeding records of Icelandic horses to challenge the hypothesis of roan being lethal in utero under homozygous condition. The roan to non-roan ratio of foals from roan × roan matings revealed homozygous roan Icelandic horses to be viable. Even though roan is known to be inherited in a dominant mode and epistatic to other coat colors, the causative mutation is still unknown. Nevertheless, an association between roan phenotype and the *KIT* gene was shown for different horse breeds. In the present study, we identified *KIT* variants by Sanger sequencing, and show that *KIT* is also associated with roan in the Icelandic horse breed.

## 1. Introduction

**Figure 1:**
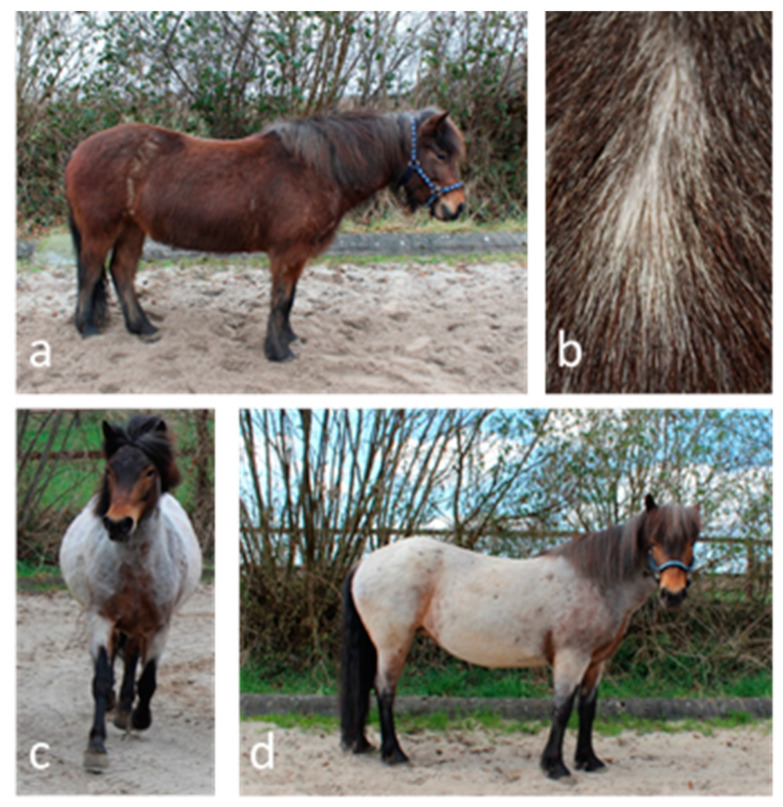
Phenotype of roan Icelandic horse (**a**–**d**): Phenotype of roan coat color of an Icelandic horse. All photographs show the same horse. (**a**) Roan horse during winter: Colored covering coat hides white undercoat. (**b**) Covering coat pushed aside, undercoat is visible. (**c**) Characteristic inverted ‘V’ on the foreleg. (**d**) Roan horse during spring: when the covering coat is shed, the white undercoat becomes visible on the horse′s body, with lower legs and head unaffected.

The coat color roan (Rn) describes a mixture of white and colored hair on the body [1], while mane, tail, head and distal legs are not affected [2,3]. Due to the similar phenotype, it can potentially be confused with other coat colors, e.g. gray [2,4], though in contrast to gray horses that are born any solid color, true roan foals are born roan. Furthermore, roan horses do not become progressively lighter in color as they age [2,3,4]. What is unique about the phenotype is the seasonal change of color: because of the undercoat hair’s being white, the horse′s body appears silver gray to white when the covering coat is shed in spring and fall. Therefore, roan foals are usually only recognized when shedding for the first time. Very characteristic is the inverted ‘V’ on the foreleg between the colored distal leg and the lighter proximal leg (Figure 1). The extent and amount of white hair can vary individually.

Roan is dominantly inherited and epistatic to other coat colors, i.e., roan combines with any basic coat color to produce various shades of roan pattern [1,4,5]. Via linkage analysis, it was shown that Rn is part of equine Linkage group II. This linkage group is located on equine chromosome 3 (ECA 3) and consists of three coat color genes, including roan, Tobiano spotting pattern and Extension locus (E), and three serum protein loci, encoding for serum albumin (Al), serum esterase (Es) and vitamin D binding protein (Gc) [3,4,5]. Even though the causative mutation for Rn is still unknown, *KIT* on ECA 3 is a major candidate gene. *KIT* is encoding for the mast cell growth factor receptor and the region is homologous to sections of human chromosome 4, mouse chromosome 5 and pig chromosome 8, which all encode for the mast cell growth factor [5]. Mutations in this region cause several white spotting phenotypes in horses, like Tobiano, Sabino, and the wide spectrum of White Spotting (Dominant White) [6,7,8,9], while phenotypic similarities like pigmentation disorders are reported in humans and mice [10,11,12,13].

Marklund et al. (1999) conducted an association analysis between Rn phenotype and variants in the *KIT* gene for roan and non-roan horses belonging to different breeds. Variants were found in several *KIT* exons. One of these variants, an exonic synonymous substitution, showed significant linkage disequilibrium (LD) to roan in numerous breeds [5]. A more recent genome-wide association study in Noriker horses identified small indels in the downstream and intronic regions of *KIT* associated with Rn [14]. Nevertheless, the genetic background of Rn remains unclear.

A survey on Belgian Draught foals registered within the Belgian Register (US) in 1937 analyzed matings of roan horses and the color ratio of their offspring. While roan × non-roan matings resulted in a ratio close to 1:1 roan to non-roan, which was to be expected for a dominant trait, the roan × roan matings showed a ratio of 1.94:1 roan to non-roan. A dominant trait with no pleiotropic effect would result in a 3:1 roan to non-roan distribution. Therefore, the authors concluded that roan is homozygous-lethal in utero, as no aborts were reported [2]. However, Sponenberg and Bellone, who postulated the existence of living homozygous Rn horses, contradicted this hypothesis [4,15]. Additional contradictory evidence, i.e., the existence of homozygous roan stallions with many roan offspring, is also emerging for other horse breeds, i.e., Noriker and Hokkaido Native horses [14,16,17].

In this study, we analyzed breeding records available in the international database Worldfengur, to investigate if the hypothesis of Rn being lethal could also be disputed for the Icelandic horse. Additionally, we sequenced the exons of the *KIT* gene in roan and non-roan Icelandic horses, and searched for Icelandic horse-specific variants associated with the coat color Rn.

## 2. Materials and Methods

### 2.1. Coat Color and Breeding Records

For this study, we obtained phenotypic data and breeding records from Worldfengur (https://www.worldfengur.com/), which is an international database and studbook for Icelandic horses. Data entry differs between countries, nevertheless, in most countries one or several national breeding associations are in charge of registration. In addition, registrars in different nations administrate the breeders′ and owner′s requests. We analyzed all breeding records of roan horses mated to roan and non-roan horses (total *n* = 3862; Rn × non-roan *n* = 3795; Rn × Rn *n* = 67) that were registered before the end of 2019, and calculated the ratios of roan to non-roan horses in the offspring. Significance levels were calculated using a Chi-Square test, with the null hypothesis that observed ratios do not differ from the expected ratios, with Rn being lethal in the homozygous state. A *p*-value < 0.05 was considered statistically significant. For breedings of roan to non-roan horses we only included matings of horses producing at least 2 offspring.

### 2.2. KIT Variants

#### 2.2.1. Animals

We obtained mane hairs of roan (*n* = 30) and non-roan Icelandic horses (*n* = 23) from Germany. Due to the fact that roan is a rare coat color and only a couple of horses contribute to the German roan Icelandic horse population, horses in the Rn group were related (up to the sixth generation). In total four roan families were present in the case group. The group of non-roan horses contained closely related (one parent and offspring trio; siblings) and unrelated horses. All horses are registered in Worldfengur and 28 of the roan Icelandic horses are also represented in our breeding analysis. The phenotypes were validated personally by one of the authors, or with the help of photographs. In addition, we checked pedigree information for plausibility to avoid misclassification of the coat color. Genomic DNA was isolated from hair samples by applying a modified protocol according to Miller et al. [18].

#### 2.2.2. KIT Gene Sequencing

For variant analysis, we amplified PCR products of 21 *KIT* exons (NM_001163866.2) including intron–exon boundaries and up to 300 bp down- and upstream. Primers were designed using Primer3 (http://bioinfo.ut.ee/primer3-0.4.0/). Primer sequences were listed in Supplementary Table S1. PCR conditions were as follows: initial activation for 3 min at 95 °C, followed by 40 cycles of 30 s at 95 °C, annealing for 1 min at the appropriate annealing temperature (Table S1) and extension for 90 s at 72 °C. PCR products were visualized on a 1.2% agarose gel. The subsequent re-sequencing of the PCR products was performed after PCR clean-up using rAPid alkaline phosphatase (Roche) and exonuclease I (New England Biolabs). We used both PCR primers with the ABI BigDye Terminator Sequencing Kit 3.1 (Applied Biosystems) to sequence PCR products on an ABI 3130. Sequence data were analyzed with Sequencher 5.0 (GeneCodes).

#### 2.2.3. Association Analysis

To investigate whether identified *KIT* variants were associated with the Rn phenotype we carried out a case-control analysis applying the option assoc in PLINK v1.07 (http://pngu.mgh.harvard.edu/purcell/plink/, [19]). We only considered variants that were genotyped in at least 90% of the animals and had a minor allele frequency > 0.01. We also excluded individuals that had a genotyping rate < 85%. Corrected empirical *p*-values were determined applying the permutation procedure (mperm) implemented in PLINK, with 10,000 permutations, to correct for multiple testing. Variants with a *p*-value < 0.05 were considered statistically significant.

## 3. Results

### 3.1. Roan to Non-Roan Ratio

Worldfengur comprises information on more than 500,000 Icelandic horses in 30 countries. The total amount of roan horses in all registered Icelandic horses is approximately 0.5% (*n* = 2494). In 2017, 2018 and 2019, around 2% of all foals were born out of matings with at least one roan parent. Out of the total number of newborn foals, about 1% were roan. The breeding data analysis showed a 0.87:1 roan to non-roan ratio for roan × non-roan matings (*n* = 3795) while roan × roan matings (*n* = 67) resulted in a 4.6:1 ratio of roan to non-roan foals. For roan × non-roan matings, we would expect a ratio of 1:1 roan to non-roan offspring. The expected ratio for roan × roan matings under the assumption of Rn being lethal in the homozygous state is 2:1 roan to non-roan offspring. Both observed ratios differ significantly from the expected ratio (Table 1).

### 3.2. Candidate Gene Analysis

All coding exons, and up to 300 bp down- and upstream of intron–exon boundaries, were re-sequenced for 30 roan and 23 non-roan Icelandic horses. In total, we identified 16 variants: 12 in intronic regions, 2 variants were located in exon 20, 1 in exon 14 and 1 in exon 21. Of the 12 intron variants, 4 were found within a 100-bp distance of the subsequent exons (Figure S1). Three variants have not been described before, and could not be found in the European Variation Archive (Table 2). Three of the exonic variants were non-synonymous, presumably leading to an amino acid exchange in the KIT protein (Table 2). None of the identified variants were exclusively present in roan horses.

### 3.3. Association Analysis

We performed an association analysis for the 16 identified *KIT* polymorphisms, using 30 roan horses as a case group and 23 non-roan horses as control. Three variants failed the missingness test and six horses (cases *n* = 3; controls *n* = 3) had a genotyping rate < 85%. Therefore, 13 variants and 47 (cases *n* = 27, controls *n* = 20) remained for association analysis. Eight variants showed a statistically significant (*p*-value < 0.05) association with the roan phenotype after permutation testing (Table 2). An insertion in intron 13 (ECA3: 79,548,356) showed the highest significance (*p*-value = 0.0002) for association.

## 4. Discussion

The Icelandic horse is a colorful breed, in which almost all coat colors are accepted and present. Therefore, the Icelandic horse breed is an adapted model for coat color genetics. Roan is a rare coat color and is also present in other breeds, e.g., Quarter Horses, Paints, Noriker, Belgian Draught horses and Shetland Ponies [15,20]. Even though it is known to be dominant and epistatic to other coat colors, the genetic background remains unclear.

A particular difficulty when dealing with roan is the indubitable identification: roan could easily be confused with gray [20]. Additionally, similar color patterns, with white hair intermingling with colored hair, exist, e.g., rabicano or roaning/roaned [15], vanish roan caused by the leopard complex [21,22], Sabino [23] and other *KIT-*mutations [9], even though most of them have not been reported in the Icelandic breed so far. It is quite challenging to identify a roan foal correctly, as their white undercoat is mostly invisible in the foal coat until their first shedding.

Misclassification of coat color could also happen during registration of the horse, since many horses are being registered by breeders, breeding organizations and owners without any verifiable color information. A large portion of the horses in Worldfengur was registered retrospectively. Worldfengur offers a plausibility check based on the dam′s and sire′s coat color, but it is not used routinely. Therefore, misclassification of coat color phenotypes cannot be excluded. 

Another source for coat color misclassification is wrong designation of maternity or/and paternity. In Holstein Friesian and other cattle breeds, 3–23% of the sire information is incorrect [24,25,26,27], and in horses parentage mistakes are also reported [28,29]. Potential mistakes in breeding records, misclassification of the coat color and the limited number of roan horses born could explain the distribution of the expected roan to non-roan ratio of 1:1 for a dominant trait, even though we tried to minimize the effect of errors by including horses with at least two offspring for Rn × non-Rn matings. However, our analysis of breeding records show that roan seems not to be lethal in the homozygous state in the Icelandic horse. Hintz and van Vleck (1979) postulated that homozygous Rn was lethal in utero in Belgian draught horses, according to offspring (*n* = 582) ratios from 1937 [2]. Nevertheless, homozygous roan horses, born before 1937 and after, were reported [30,31,32]. In addition, the existence of homozygous roan stallions with many roan offspring was shown for the Quarter horse and the Hokkaido Native horse [16,17]. This is in accordance with a previously published report of a homozygous Rn Noriker horse by Grilz-Seger et al. (2020) [14]. One reason for the contradicting reports could be the misclassification of the coat color phenotype. Another reason could be that the allele causing roan is different between breeds. For instance, breed-specific segregation of coat color alleles is known for chestnut (*MC1R*) [33], White spotting (*KIT*) [34] and Sabino [7,35].

Association studies have been very successful in uncovering causative variants for coat color phenotypes in different species [36,37,38]. The power of association studies depends not only on sample size and on the number of markers genotyped, but also on the case-to-control ratio, the causative allele frequency, the mode of inheritance, LD between markers, and the prevalence and penetrance of the phenotype. These factors determine the probability of detecting a putative causative variant [39]. In our study, the number of well-characterized cases and controls limits sample sizes. Additionally, we included horses that were distantly related to each other, and therefore are likely to introduce a cryptic population structure, which can increase the likelihood of false positives. Furthermore, our association analysis only included 16 variants, which were not equally distributed across the *KIT* gene. To avoid misclassification of the phenotypes of our cases and controls, one of the authors validated the coat color personally, or with the help of photographs. In addition, pedigree information was checked for plausibility. Roan is a rare coat color; however, it is dominantly inherited, and therefore horses with the Rn phenotype should be either heterozygous or homozygous for the causative allele. Nevertheless, results of the association analysis should be validated in a larger cohort of unrelated samples. 

The *KIT* gene is associated with a wide variation of coat color phenotypes [6,7,8,9], and has been shown to be associated with roan in other horse breeds [4,5,14]. Marklund et al. (1999) reported a variant in *KIT* exon 19 that was associated with roan coat color in different horse breeds (Figure S1) [5], whereas Grilz-Seger et al. (2020) identified 3 *KIT* variants in complete linkage disequilibrium with a variant identified via a genome-wide association study (Figure S1) and whole-genome sequencing, in Noriker, Slovenian draught horses, Quarter horses, Murgese and Belgian draught horses [14]. Interestingly, the identified association by Marklund et al. (1999) could not be shown in Gotland and Shetland ponies [5]. Furthermore, the associated variants reported by Grilz-Seger et al. (2020) were not present in roan German Warmblood horses, or Trakehner and Shetland Ponies. We demonstrated a significant association of *KIT* with roan coat color in the Icelandic horse. However, we did not identify the same variants reported by Marklund et al. (1999) or Grilz-Seger et al. (2020) [5,14], even though the variants are in close proximity to each other. Our data, together with the former reports, suggest allelic heterogeneity of roan in horses. As Grilz-Seger et al. (2020) suggested, this might be explained by the population history of horse breeds [14]. Draught horses, Thoroughbreds and Northern European Pony breeds show distinct genetic distances, so the roan phenotype might be traced back to different founder populations. Even though we have shown association between roan and *KIT* in the Icelandic horse, we cannot exclude other genetic loci causing roan.

## 5. Conclusions

In summary, we detected eight variants within the *KIT* gene that had significant association with roan in Icelandic horses. Variants in *KIT* that were reported to be associated with roan in other breeds were not found. Analysis of the breeding information for matings with at least one roan horse did not show any evidence of lethality for homozygous roan. Furthermore, it is possible that roan shows allelic heterogeneity between different horse breeds. Consequently, further research is required to identify the causative mutation(s), and to shed more light on the genetic background of the coat color roan.

## Figures and Tables

**Table 1:** Matings of roan × non-roan (≥2 offspring) and roan × roan. Significance was calculated using a Chi-Square test.

Matings	Non-Roan Offspring	Roan Offspring	Percentage	Significance (*p*-value)
roan × non-roan (*n* = 3795) ^1^	2027	1768	46.59	2.619 × 10^−5^
roan × roan (*n* = 67)	12	55	82.08	7.406 × 10^−3^

^1^ includes crossing of either roan mare or roan stallion.

**Table 2:** Identified *KIT* variants in Icelandic horses. Positions of the variants are based on EquCab3.0. The equine genome reference sequence was considered to represent the reference allele (ref). The alternate allele (alt) and the allele frequency (AF) of the alternate allele in non-roan (non-Rn) and roan (Rn) horses are given. Empirical *p*-values were derived from an association study using the options assoc and mperm implemented in PLINK.

*KIT*	Variant Position	Allele 1 (Ref)	Allele 2 (alt)	AF Allele 2 (non-Rn/Rn)	Protein (NP_001157338.2)	Accession Number ^1^	Significance (*p*–value)
Intron 3	ECA3: 79,578,812	T	C	0.66/0.00	-	rs68706118	NA ^2^
Intron 6	ECA3: 79,566,965	A	C	0.50/0.27	-	rs1140206871	NA ^2^
Intron 13	ECA3: 79,548,356	CCC	CC	0.67/0.30	-	-	0.0002
Exon 14	ECA3: 79,548,165	C	T	0.01/0.00	p.K700E	rs1142192381	1.0000
Intron 15	ECA3: 79,545,768	C	T	0.13/0.00	-	rs1142435913	NA ^2^
Intron 16	ECA3: 79,545,073	C	G	0.67/0.30	-	rs1150320794	0.0004
Intron 16	ECA3: 79,544,396	T	C	0.98/0.52	-	rs1145091198	0.4286
Intron 16	ECA3: 79,544,372	T	A	0.67/0.30	-	rs1144027176	0.0004
Intron 16	ECA3: 79,544,336	C	A	0.13/0.35	-	-	0.0286
Intron 19	ECA3: 79,540,110	T	C	0.72/0.34	-	rs394038202	0.0006
Exon 20	ECA3: 79,540,020	G	A	0.54/0.36	synonymous	rs1151991439	0.0336
Exon 20	ECA3: 79,539,989	T	C	0.54/0.25	p.I924V	-	0.0003
Intron 20	ECA3: 79,538,853	G	C	0.89/0.75	-	rs396171297	0.1108
Exon 21	ECA3: 79,538,738	C	T	0.53/0.22	p.A960P	rs1141982296	0.0003
3′ flanking	ECA3: 79,538,601	C	T	0.19/0.36	-	rs1140692936	0.1448
3′ flanking	ECA3: 79,538,561	G	A	0.22/0.38	-	rs1146165609	0.1778

^1^ variants′ accession numbers were obtained from Ensembl genome database and European Variation Archive. ^2^ NA = not available, variants did not pass analysis quality threshold.

## Supplementary Materials

The following are available online at https://www.mdpi.com/2073-4425/11/6/680/s1, Table S1: Primer sequences for amplification of *KIT* exons; Figure S1. Equine *KIT* gene structure with location of identified *KIT* variants.
