# Supplementary material for: Coat Color Roan Shows Association with KIT Variants and No Evidence of Lethality in Icelandic Horses
Source: Genes (Basel). 2020 Jun 22;11(6):680. doi: 10.3390/genes11060680 (PMC7348759; doi:10.3390/genes11060680)
Supplement: Supplementary file 1 [file genes-11-00680-s001.zip › Table S1.pdf]

Table S1. Primer sequences for amplification of *KIT* exons

Target *KIT*	Primer	Sequence	Product length	Annealing temperature
Exon 1	KIT_Ex1_F<br>KIT_Ex1_R	5'-CAACACAGGGGAGGAGATGAAGG-3'<br>5'-AAAAGCCTCTCCAACTTTGCCCT-3'	1090	63°C
Exon 2	KIT_Ex2_F<br>KIT_Ex2_R	5'-TGCATACCAAGTGTGACCCTCAG-3'<br>5'-AGTATCAGAGCCTTGCAGACCTG-3'	709	62°C
Exon 3	KIT_Ex3_F<br>KIT_Ex3_R	5'-AGCAGCCATTTGAGTCACTAGGG-3'<br>5'-GTGGGGCTGGGACTGTGAAATAA-3'	575	62°C
Exon 4	KIT_Ex4_F<br>KIT_Ex4_R	5'-GTCCAGGAGCATTAGAACCTCACA-3'<br>5'-GTTCCACCTCACAGACACACCAG-3'	525	62°C
Exon 5	KIT_Ex5_F<br>KIT_Ex5_R	5'-TGGGTGAGAACAGTGGGATGAGT-3'<br>5'-TTTGTTTGTTTGGTGGGGAGGGG-3'	959	63°C
Exon 6	KIT_Ex6_F<br>KIT_Ex6_R	5'-CCTGGGTCTGGGTATAGGCTAACT-3'<br>5'-CCCCAAACTCCCTTCCATAGCAG-3'	664	63°C
Exon 7	KIT_Ex7_F<br>KIT_Ex7_R	5'-CAAAATTGACCTTGCTGCTCGGG-3'<br>5'-CGTCTACAAGGTCACGTGGTCAG-3'	775	63°C
Exon 8	KIT_Ex8_F<br>KIT_Ex8_R	5'-GTTTGACCCCTCACATCCACCTC-3'<br>5'-TACAGCTGCATTTCCCACACACA-3'	649	63°C
Exon 9	KIT_Ex9_F<br>KIT_Ex9_R	5'-GCTTTCCTCCTGCATGCTTTTCC-3'<br>5'-TGACAGTAGAACGCAATGCAGGT-3'	798	62°C
Exon 10	KIT_Ex10_F<br>KIT_Ex10_R	5'-GGGTTTCCGTGATTGTGGTGAAG-3'<br>5'-GTAAGTGGCAGTGATGGTGAGGG-3'	646	62°C
Exon 11	KIT_Ex11_F<br>KIT_Ex11_R	5'-ATCCATCCCCACACTCTGTTCAC-3'<br>5'-TGAGCATCTTCACAGCAACAGTCA-3'	693	62°C
Exon 12 +<br>Exon 13	KIT_Ex12/13_F<br>KIT_Ex12/13_R	5'-GGAACCCCAGTAGCTTCCTTTGT-3'<br>5'-ATCCCACCCCACGATGAGAAAAC-3'	712	62°C
Exon 14	KIT_Ex14_F<br>KIT_Ex14_R	5'-CACCTGGCCCCTAATCTCTCAGA-3'<br>5'-GAATGACACCAAGAGCGGAACCA-3'	706	63°C
Exon 15	KIT_Ex15_F<br>KIT_Ex15_R	5'-CCATCTAGCTCCTGGGGATTGGA-3'<br>5'-TAACGGATGTGAACTTGCAGGCA-3'	710	63°C
Exon 16	KIT_Ex16_F<br>KIT_Ex16_R	5'-TTCAACACCCACCTTTCCTCACA-3'<br>5'-GCACTGCAACTACATGAGGTACCT-3'	615	62°C
Exon 17	KIT_Ex17_F<br>KIT_Ex17_R	5'-CTGCCCCTTAAGTGTCGTTGGTGA-3'<br>5'-TTCTGTTTCTTTCACGTGCCCCA-3'	868	63°C
Exon 18	KIT_Ex18_F<br>KIT_Ex18_R	5'-GGGTGTTTTCCTAAAGCTGCCAA-3'<br>5'-AGAAGGAAACACATCTTGGCCCT-3'	849	62°C
Exon 19	KIT_Ex19_F<br>KIT_Ex19_R	5'-CCTGTGAAATGGATGGCACCTGA-3'<br>5'-ACGATCTGCTTGAATGTTGGCCT-3'	740	63°C
Exon 20	KIT_Ex20_F<br>KIT_Ex20_R	5'-CACCTGCTGAAATGTAAGGGCCA-3'<br>5'-TCCCCTCCTCATCCCAAATCCTT-3'	689	63°C
Exon 21	KIT_Ex21_F<br>KIT_Ex21_R	5'-TTGGGTTTTGGCTGTGGTCTTGT-3'<br>5'-TTGGAAAGGACTCGGGGTGTCTA-3'	571	63°C
