# Supplementary figures and images for: Coat Color Roan Shows Association with KIT Variants and No Evidence of Lethality in Icelandic Horses
Source: Genes (Basel). 2020 Jun 22;11(6):680. doi: 10.3390/genes11060680 (PMC7348759; doi:10.3390/genes11060680)

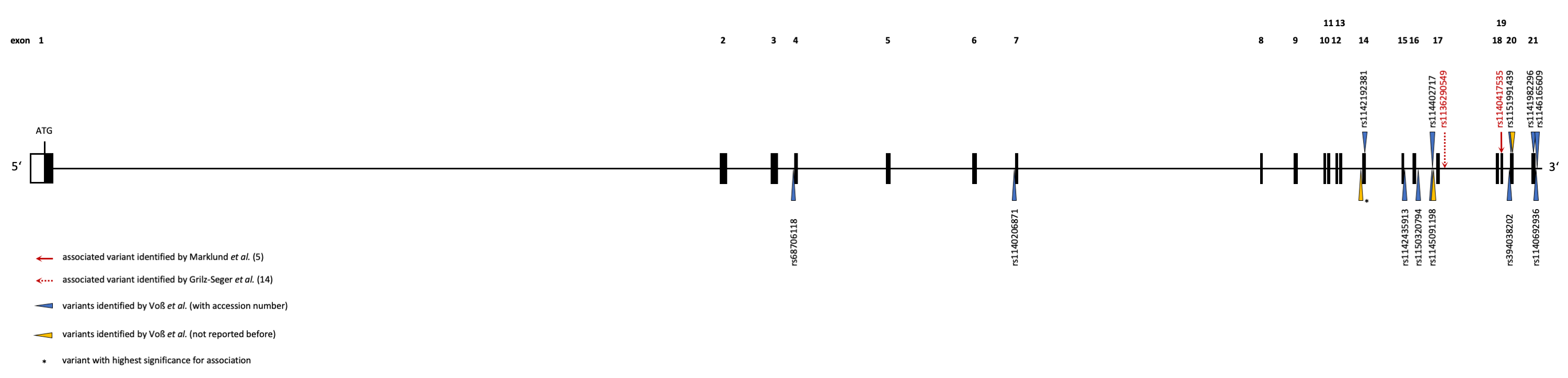

Supplement: Supplementary file 1 [file genes-11-00680-s001.zip › Figure S1.tiff]
